# Tumour DNA methylation markers associated with breast cancer survival: a replication study

**DOI:** 10.1186/s13058-024-01955-x

**Published:** 2025-01-17

**Authors:** Elaheh Zarean, Shuai Li, Ee Ming Wong, Enes Makalic, Roger L. Milne, Graham G. Giles, Catriona McLean, Melissa C. Southey, Pierre-Antoine Dugué

**Affiliations:** 1 Precision Medicine, School of Clinical Sciences at Monash Health, Monash University, 246 Clayton Road, Clayton, VIC 3168 Australia; 2 Centre for Epidemiology and Biostatistics, Melbourne School of Population and Global Health, The University of Melbourne, Parkville, VIC Australia; 3 Department of Data Science and AI, Faculty of Information Technology, Monash University, Clayton, VIC Australia; 4 Cancer Epidemiology Division, Cancer Council Victoria, Melbourne, VIC Australia; 5 Anatomical Pathology, Alfred Health, The Alfred Hospital, Melbourne, VIC Australia; 6 Department of Clinical Pathology, Melbourne Medical School, The University of Melbourne, Parkville, VIC Australia

**Keywords:** DNA methylation, Breast cancer, Survival, FFPE tissue, Prognostic factors

## Abstract

**Background:**

Tumour DNA methylation has been investigated as a potential marker for breast cancer survival, but findings often lack replication across studies.

**Methods:**

This study sought to replicate previously reported associations for individual CpG sites and multi-CpG signatures using an Australian sample of 425 women with breast cancer from the Melbourne Collaborative Cohort Study (MCCS). Candidate methylation sites (N = 22) and signatures (N = 3) potentially associated with breast cancer survival were identified from five prior studies that used The Cancer Genome Atlas (TCGA) methylation dataset, which shares key characteristics with the MCCS: comparable sample size, tissue type (formalin-fixed paraffin-embedded; FFPE), technology (Illumina HumanMethylation450 array), and participant characteristics (age, ancestry, and disease subtype and severity). Cox proportional hazard regression analyses were conducted to assess associations between these markers and both breast cancer-specific survival and overall survival, adjusting for relevant participant characteristics.

**Results:**

Our findings revealed partial replication for both individual CpG sites (9 out of 22) and multi-CpG signatures (2 out of 3). These associations were maintained after adjustment for participant characteristics and were stronger for breast cancer-specific mortality than for overall mortality. In fully-adjusted models, strong associations were observed for a CpG in *PRAC2* (per standard deviation [SD], HR = 1.67, 95%CI: 1.24–2.25) and a signature based on 28 CpGs developed using elastic net (per SD, HR = 1.48, 95%CI: 1.09–2.00).

**Conclusions:**

While further studies are needed to confirm and expand on these findings, our study suggests that DNA methylation markers hold promise for improving breast cancer prognostication.

**Supplementary Information:**

The online version contains supplementary material available at 10.1186/s13058-024-01955-x.

## Introduction

Breast cancer is a heterogeneous disease characterised by a wide range of alterations in the genomic, epigenomic, transcriptomic, and proteomic landscape of cancer cells [1, 2]. These alterations have the potential to meet the important need of identifying prognostic molecular markers, beyond traditional clinicopathological variables, but are seldom used in the clinic. Epigenetic changes are a ‘hallmark of cancer’ and have been widely reported in breast tumours [3]. Such changes include DNA methylation alterations occurring in tumour tissue compared with normal breast tissue as well as DNA methylation differences by key clinical variables such as hormonal receptor status, tumour stage, treatment modality or age at diagnosis [4–7]. DNA methylation is a fairly stable epigenetic modification. Modern micro-assays enable DNA methylation measurement at hundreds of thousands of sites across the genome. This makes DNA methylation a promising molecular marker for precision medicine approaches to breast cancer outcome prediction [3]. However, there is a relative lack of large genomic datasets of breast tumour tissues with DNA methylation measurements and outcome follow-up. The main publicly available resource, The Cancer Genome Atlas (TCGA) [8], has been used extensively to investigate a wide range of research questions. Among these, several studies have reported associations with breast cancer survival for individual CpG sites [9, 10], and/or developed epigenetic scores that predict outcomes, with or without replication in external small datasets [11–15]. To our knowledge, these findings have not received major attention and no studies have been conducted to confirm their potential clinical relevance through independent replication. Another challenge in considering the results from these studies is that they have used different methodological approaches, e.g. in terms of adjustment variables [9, 10, 13] or filtering of CpGs [11, 12, 14–16] tested for association with survival.

In this study, we considered several studies that have reported individual CpG sites or multi-CpG signatures in breast tumours to be associated with breast cancer survival using the TCGA DNA methylation dataset, and assessed their replication using a breast tumour dataset with similar characteristics in terms of sample size, tissue (formalin-fixed paraffin-embedded [FFPE]), technology (Illumina HumanMethylation450 array) and participant characteristics such as age, ancestry and disease subtype and severity.

## Material and methods

### Study sample

The Melbourne Collaborative Cohort Study (MCCS) is a cohort of 41,513 participants of white European ancestry aged 40–69 years when recruited in 1990–1994 [17]. Incident cancer cases were identified via linkage with the Victorian Cancer Registry. Breast tumours stored as FFPE (formalin-fixed paraffin-embedded) tissues were retrieved from diagnostic laboratories and reviewed by qualified pathologists [18]. Following pathologist evaluation, all FFPE tissues were sectioned at 3 µm thick. A subset of these sections underwent hematoxylin and eosin (H&E) staining for microscopic examination, while the remaining sections were left unstained. All samples were desiccated and stored at 4 °C for up to 15 years [18, 19]. Linkage with the Victorian Registry of Births, Deaths and Marriages and the National Death Index was used to ascertain deaths, including breast cancer-specific deaths [17]. Immunohistochemical staining and breast cancer subtyping were performed using the methods outlined by Blows et al. [20].

## Genome‑wide DNA methylation profiling

The HumanMethylation450K (HM450K) BeadChip array was used to measure methylation in DNA extracted from FFPE tumour sections. The full protocol, detailed in Wong et al. [18], is a modification of the standard HM450K protocol for FFPE samples and incorporates an additional quality control (QC) step. Briefly, out of 474 FFPE breast tumour samples, 430 (90%) successfully passed the three mandatory QC checkpoints, which assessed DNA quality (Qubit, Life Technologies), and the presence of DNA following bisulfite conversion and restoration (qPCR with an in-house assay). The *ENmix* pipeline was used to pre-process and normalise the methylation data, which includes background correction, dye-bias correction, and probe-type bias correlation, and has excellent reported performance [21]. Samples and probes were excluded if the detection P-value was greater than 0.01 for over > 5% of the samples or probes respectively. The final methylation dataset consisted of 425 samples, after exclusion of 5 samples with missing data for stage and molecular subtypes define by immunohistochemistry markers (IHC-based), and 476,155 CpG sites. Beta-values, ranging from 0 to 1, were used to represent the methylation percentage at each CpG site.

## Candidate studies

We searched the literature for studies that stored breast tumours as FFPE sections, measured DNA methylation using the HM450K assay, and carried out an epigenome-wide association study (EWAS) of, or developed a methylation-based risk score for either overall or breast-cancer specific survival. The following studies were retained, all of which used TCGA data:

(i) Kim et al. [10] used 692 breast tumours from TCGA as the discovery set (cause-specific death N = 87) and 180 breast tumours (GSE72308, cause-specific death N = 29) as the replication set. From six EWASs carried out in the discovery dataset for breast cancer overall and three gene expression-based subtypes (luminal A, luminal B, basal-like), 20 associations were identified (P < 1 × 10^–7^, 17 unique CpGs) with little evidence of replication.
(ii) de Almeida et al. [9] used 780 breast tumours from TCGA and pre-selected CpGs based on their correlation with gene expression, and differential methylation between tumour tissue and normal adjacent samples. The authors reported nominally significant associations (P < 0.05) for 5 CpGs using unadjusted Cox models. No validation analysis was conducted.
(iii) Tao et al. [12] used data from 788 TCGA breast tumours; they pre-selected CpGs based on differential methylation between normal and tumour tissue and nominal associations with survival, after which they applied stepwise regression to derive a 7-CpG mortality risk score. No validation analysis was conducted.
(iv) Du et al. [11] used a similar approach to Tao et al. [12] to pre-select CpGs (N = 1,777), from which they used penalised regression (least absolute shrinkage and selection operator, LASSO) to develop a score based on 16 CpGs. Validation was carried out in an external set of 62 women, with a reported AUC of 0.94.
(v) Liu et al. [15] also used regularised regression (elastic net) to derive a 28-CpG signature of survival using a discovery set including 60% TCGA data. This signature was validated in a separate internal TCGA validation set (40% of the total data) and several external datasets, with AUCs ranging from 0.62 to 0.65 at various follow-up time points.

Other studies considered, but not included were: Hao et al. [16], which was similar to Du et al. [11] and Liu et al. [15], but did not provide the specific regression coefficients (weights) for each CpG site; Peng et al. [14] which focused on triple-negative tumours, hence with small number of deaths in their study and ours; Pedersen et al. [13] investigated associations with survival between two groups before and after neoadjuvant chemotherapy treatment, for which we did not have data available.

## Statistical analysis

There were no overlapping CpGs across the five studies included in the analyses. As the included studies used different variables for adjustment, we carried out the analysis using four Cox proportional hazard regression models for overall and breast cancer-specific survival: Model 0: unadjusted [9, 11, 15] Model 1: adjusted for age at diagnosis and country of birth; Model 2: Model 1 + additional control for IHC-based subtypes and stage, using stratified Cox models. "Stratified" refers to the model allowing the baseline hazard rate to vary by the stratification variable while assuming a common HR for association of methylation with survival across strata [22]; Model 3: Model 2 + additional adjustment for tumour purity [10]. For overall survival models, heterogeneity between subtypes was investigated using a likelihood ratio test for the interaction between DNA methylation and subtype. To align with Kim et al. [10] for replication purposes, subgroup analyses were carried out for luminal A and luminal B subtypes. Additionally, we assessed the heterogeneity between subtypes by testing interactions between tumour subtype and DNA methylation using likelihood ratio tests. Tumour purity was estimated using the R function *InfiniumPurify* [23]. As the directions and effect sizes for associations in De Almeida’s study were not reported [9], we calculated them using the TCGA data. Multiple testing was considered but not explicitly accounted for. All analyses were performed using R version 4.3.0.

## Results

Of 425 women included in the analysis, 168 died (follow-up time; median (interquartile range (IQR)): 15.5 (12–19.4) years, follow-up until 2019), including 66 of breast cancer (follow-up time, median (IQR): 12.1 (9.2–15.8) years, follow-up until 2015). Luminal A and B tumours represented 77% of all tumours (242 and 87, respectively), HER2-positive 7% (N = 31) and triple-negative 15% (N = 65). The estimated tumour purity of the samples was 0.63 (IQR: 0.55–0.70), similar to other studies [10, 12, 24], Table 1.

**Table 1:** Clinical characteristics of the study sample (Melbourne Collaborative Cohort Study, N = 425 women)

Clinical characteristics		N women (%)	All-cause death (%)	Breast cancer death (%)
		425 (100)	168 (40)	66 (16)
IHC-based subtype	Luminal A	242 (57)	101 (42)	33 (14)
	Luminal B	87 (20)	32 (37)	12 (14)
	HER2-positive	31 (7)	10 (32)	7 (23)
	Triple-negative	65 (15)	25 (38)	14 (22)
Stage	I	246 (58)	79 (32)	14 (6)
	II	135 (32)	57 (42)	28 (21)
	III/IV	44 (10)	32 (73)	24 (55)
Country of birth	Aust/NZ/Other	344 (81)	136 (40)	52 (15)
	Northern Europe	20 (5)	9 (45)	5 (25)
	Southern Europe	61 (14)	23 (38)	9 (15)
Age, median (IQR)^1^		64 (57–70)		
Tumour purity, median (IQR)		0.63 (0.55–0.70)		

^1^Median (Interquartile range: Q1-Q3)

## Replication of associations at individual CpGs

For De Almeida et al. [9], 3 of 5 CpGs showed evidence of associations with breast cancer-specific survival that were consistent in direction (P ≤ 0.01, HRs per SD between 1.41 and 1.58 for *HOXD9*, *C17orf93* and *TDRD10*) in Model 0 used by the authors, Table 2. For the two remaining CpGs, the HRs were consistent in direction with De Almeida et al.’s [9] study (cg04475027, HR = 1.26, P = 0.08, cg01268824, HR = 1.18, P = 0.18), Table 2. Only cg12374721 (*C17orf93*) retained a clear association in adjusted models (Model 3, HR = 1.67, 95%CI: 1.24–2.25, P = 0.001), Table 2. The results for overall survival were consistent with breast cancer-specific survival, with smaller HRs (Table S1).

**Table 2:** Associations of tumour DNA methylation with breast cancer-specific survival for 22 individual CpG sites using MCCS data (N cases = 425, N breast cancer deaths = 66)

Study	CpG	Gene		Model 0^a^		Model 1^a^		Model 2^a^		Model 3^a^		Evidence of replication
			Direction	HR, 95%CI	P	HR, 95%CI	P	HR, 95%CI	P	HR, 95%CI	P	
De Almeida et al. [9]	cg01268824	*ZNF154*	+	1.18 (0.93,1.51)	0.18	1.17 (0.91,1.49)	0.22	1.08 (0.83,1.41)	0.55	1.06 (0.79,1.42)	0.69	No
	cg22674699	*HOXD9*	+	1.46 (1.11,1.91)	0.01	1.45 (1.10,1.91)	0.01	1.24 (0.93,1.65)	0.14	1.24 (0.91,1.67)	0.17	Yes
	cg12374721	*C17orf93*	+	1.58 (1.21,2.07)	0.001	1.58 (1.21,2.06)	0.001	1.63 (1.23,2.15)	0.001	1.67 (1.24,2.25)	0.001	Yes
	cg18081940	*TDRD10*	+	1.41 (1.08,1.85)	0.01	1.40 (1.07,1.84)	0.02	1.24 (0.93,1.64)	0.14	1.24 (0.91,1.69)	0.17	Yes
	cg04475027	*TMEM132C*	+	1.26 (0.97,1.63)	0.08	1.25 (0.96,1.62)	0.10	1.06 (0.82,1.38)	0.66	1.04 (0.79,1.37)	0.77	No
Kim et al. [10]	cg03985718	*TGFBRAP1*	−	1.16 (0.90,1.51)	0.25	1.16 (0.89,1.50)	0.28	1.18 (0.90,1.54)	0.22	1.18 (0.90,1.54)	0.23	No
	cg04921068	*PPM1L*	−	0.86 (0.65,1.13)	0.28	0.87 (0.66,1.15)	0.31	0.85 (0.66,1.10)	0.22	0.86 (0.66,1.12)	0.26	No
	cg15462203	*DVL1*	−	0.92 (0.74,1.15)	0.46	0.92 (0.74,1.15)	0.46	0.99 (0.80,1.23)	0.94	1.00 (0.80,1.24)	0.99	No
	cg17827670	*AHCYL2*	−	0.64 (0.46,0.90)	0.01	0.65 (0.46,0.91)	0.01	0.66 (0.47,0.92)	0.01	0.66 (0.47,0.93)	0.02	Yes
	cg09926728	*SH3PXD2A*	−	0.86 (0.71, 1.03)	0.11	0.86 (0.72,1.04)	0.13	0.80 (0.64,0.98)	0.04	0.80 (0.64,1.00)	0.05	Yes
	cg18703983	*KCNS3*	−	0.74 (0.62, 0.89)	0.001	0.74 (0.61,0.89)	0.002	0.77 (0.62,0.96)	0.02	0.77 (0.62,0.96)	0.02	Yes
	cg16976520	*ESYT2*	−	0.89 (0.71, 1.11)	0.29	0.88 (0.71,1.10)	0.28	0.87 (0.70,1.09)	0.24	0.88 (0.70,1.10)	0.26	No
	cg17735983	*MZF1*	+	1.34 (1.14, 1.59)	0.001	1.34 (1.13,1.59)	0.001	1.26 (1.06,1.50)	0.01	1.26 (1.05,1.51)	0.01	Yes
	cg10678486	*ELAC1*	+	1.18 (1.01, 1.38)	0.04	1.19 (1.01,1.40)	0.04	1.24 (1.04,1.47)	0.01	1.24 (1.04,1.47)	0.02	Yes
	cg13447284		−	1.08 (0.84,1.39)	0.54	1.09 (0.84,1.40)	0.52	1.14 (0.89,1.47)	0.29	1.16 (0.89,1.50)	0.27	No
	cg24328142	*TSPAN15*	−	0.76 (0.62,0.95)	0.01	0.750.61,0.93)	0.01	0.78 (0.63,0.97)	0.02	0.77 (0.62,0.95)	0.02	Yes
	cg03216043	*DNM2*	−	1.09 (0.84,1.40)	0.52	1.08 (0.83,1.39)	0.57	1.04 (0.80,1.34)	0.78	1.02 (0.78,1.34)	0.87	No
	cg22776912	*TMC3*	−	1.01 (0.79,1.29)	0.94	1.00 (0.78,1.28)	0.98	1.06 (0.79,1.44)	0.69	1.06 (0.79,1.43)	0.70	No
	cg06956006	*ACLY*	−	0.99 (0.78,1.25)	0.91	0.97 (0.76,1.24)	0.82	0.96 (0.74,1.24)	0.74	0.96 (0.74,1.25)	0.78	No
	cg00175150	*ECM1*	−	0.84 (0.70,1.01)	0.07	0.84 (0.70,1.01)	0.07	0.92 (0.74,1.14)	0.44	0.91 (0.74,1.13)	0.40	No
	cg15348839		−	0.98 (0.78,1.24)	0.88	0.98 (0.78,1.24)	0.88	0.97 (0.77,1.23)	0.83	0.98 (0.77,1.25)	0.88	No
	cg12511487		−	0.81 (0.66,1.00)	0.05	0.82 (0.66,1.01)	0.06	0.83 (0.66,1.05)	0.12	0.81 (0.64,1.03)	0.09	No

^a^Model 0: unadjusted; Model 1: adjusted for age and country of birth; Model 2: stratified for stage (I; II; III/IV) and IHC-based subtype (luminal A, luminal B, HER2-positive, triple-negative) + adjusted for age and country of birth; Model 3: Model 2 + additional adjustment for tumour purity

Most (76%) of the associations for the 17 CpGs identified by Kim et al. [10] were in the same direction in our study, with evidence of replication at P < 0.05 (Model 3, cause-specific survival) for six CpGs: cg17827670 (*AHCYL2,* per SD, HR = 0.66, P = 0.02), cg18703983 (*KCNS3*, HR = 0.77, P = 0.02), cg17735983 (*MZF1*, HR = 1.26, P = 0.01), cg10678486 (*ELAC1*, HR = 1.24, P = 0.02), cg24328142 (*TSPAN15*, HR = 0.77, P = 0.02), and cg09926728 (*SH3PXD2A*, HR = 0.80, P = 0.05), Table 2. The results for overall survival were consistent with cause-specific survival, with weaker associations, Table S1. Heterogeneity by subtype was only detected for cg17735983 (*MZF1*, P = 0.04). This association was also detected for luminal B tumours (HR = 1.33, P = 0.01), Table S3.

In the unadjusted model, the 16-CpG and 28-CpG scores showed a fairly strong association with cause-specific survival (HR = 1.41, P = 0.002 and HR = 1.62, P = 5.5 × 10^–5^), Table 3, and weaker associations with overall survival (HR = 1.26, P = 0.02 and HR = 1.34, P = 1 × 10^–4^), Table S2. These associations were slightly attenuated after adjustment for clinical variables with the 16-CpG (HR = 1.17, P = 0.03) and 28-CpG signatures (HR = 1.23, P = 0.007) though retained association with overall survival, Table S2, and cause-specific survival (HR = 1.40, P = 0.003 and HR = 1.16, P = 8.8 × 10^–5^), Table 3.

**Table 3:** Associations of three tumour DNA methylation-based signatures with breast cancer-specific survival using MCCS data (N cases = 425, N breast cancer deaths = 66)

Methylation-based predictors of survival	Breast cancer-specific survival; N = 425; N deaths = 66							
	Model 0^a^		Model 1^a^		Model 2^a^		Model 3^a^	
	HR, 95%CI	P	HR, 95%CI	P	HR, 95%CI	P	HR, 95%CI	P
Du et al. [11]; 7 CpGs	1.12 (0.88,1.42)	0.37	1.22 (0.88,1.42)	0.35	0.97 (0.75,1.26)	0.83	1.00 (0.76,1.31)	0.97
Tao et al.[12]; 16 CpGs	1.41 (1.13,1.75)	0.002	1.40 (1.12,1.75)	0.003	1.22 (0.98,1.53)	0.08	1.22 (0.96,1.55)	0.10
Liu et al.[15]; 28 CpGs	1.62 (1.28,2.05)	5.5 × 10^–5^	1.61 (1.27,2.05)	8.8 × 10^–5^	1.40 (1.08,1.83)	0.01	1.48 (1.09,2.00)	0.01

^a^Model 0: unadjusted; Model 1: adjusted for age and country of birth; Model 2: stratified for stage (I; II; III/IV) and IHC-based subtype (luminal A, luminal B, HER2-positive, triple-negative) + adjusted for age and country of birth; Model 3: Model 2 + additional adjustment for tumour purity

## Discussion

Most studies that have investigated tumour DNA methylation markers of breast cancer survival have been limited by the small number of events and lack of independent replication. We sought to replicate findings from five previous studies that produced non-overlapping lists of prognostic CpGs. We found: (i) some evidence of replication for both individual CpGs and multi-CpG signatures; (ii) the majority of associations were stronger for cause-specific mortality, which may indicate that they were not false-positives.

The number of cause-specific deaths in TCGA is at most 101, so our study including 66 cause-specific deaths and 168 for all-cause mortality is a valuable addition. These are nevertheless small sample sizes and only allow detection of relatively large effects, in particular when considering a strict Bonferroni correction threshold for the HM450 assay of 1 × 10^–7^, as in Kim et al. [10]. Factors such as menopausal status and race were considered in their study [10] as these might influence the association between DNA methylation and survival, but we did not undertake these analyses as we did not have detailed data on these variables; most of our participants were post-menopausal and all women in our cohort were of White European origin. Additional subgroup analyses were not conducted, as they were anticipated to be underpowered and unlikely to yield robust conclusions. Kim et al. [10] reported minimal evidence of replication in a small external dataset, whereas we were able to corroborate their findings for at least 6 CpGs in *AHCYL2*, *SH3PXD2A*, *KCNS3, MZF1, ELAC1* and *TSPAN15*. Most other HRs were in the same direction, which suggests additional studies with larger sample sizes might more clearly identify survival-associated CpGs.

Therefore, while further studies are required to confirm and extend these findings, it seems likely that tumour DNA methylation markers identified from epigenome-wide association studies may have a role for breast cancer prognostication.

Beyond clinical characteristics, methodological variations in pre-processing, normalization of methylation data, and subtype definition (IHC- vs gene expression-based) can further contributing to lack of replication across studies. Even when using the same dataset (TCGA), discrepancies in statistical approaches have led to different conclusions. For example, in the studies we considered, three used regularised regression but with different models: two used LASSO [11, 16], which selects a smaller, more relevant subset of CpGs by shrinking the estimates of weaker predictors to zero, while the other one used elastic net [15], which combines this selection process with a more moderate reduction in the influence of predictors that contribute less to the outcome. Another study based on TCGA used backward selection [11] of CpGs, which is considered an inferior method to derive a prediction model, and the resulting association with breast cancer survival was close to null in our study.

Using different statistical approaches and adjusting for different clinical characteristics to develop risk scores resulted in inconsistent predictive accuracy in previous studies based on the TCGA dataset. Hao et al. [16] developed mortality risk scores for various cancers, including breast cancer, and reported c-indices of 0.61–0.63 in their internal replication set. Liu et al. [15] used a similar approach to derive a methylation-based predictor of mortality, reporting AUCs ranging from 0.62 to 0.75 in the discovery set and from 0.62 to 0.65 in the validation set at various follow up times. In contrast, Du et al. [11] reported an implausibly high AUC of 0.94, raising concerns about potential overfitting when applied to the validation set. Despite the use of similar datasets, none of these studies provided the respective weights necessary for replication, limiting the ability to verify their findings. In our study, although the associations between individual CpGs and multi-CpG signatures with cause-specific survival and overall survival were somewhat attenuated after adjusting for clinical characteristics, they remained robust overall. This consistency suggests that DNA methylation plays a role in prognosis, independent of hormonal factors, tumour subtype, or stage.

The strongest association we observed was for *C17orf93* also known as *PRAC2* or *HOXB13-AS1,* which is highly expressed in prostate, rectum, colon and is a potential prostate cancer susceptibility gene [25–27]. To our knowledge it has not been reported in the field of breast cancer and was ranked at the bottom of the ‘OncoScore’ list used by De Almeida et al. [9]. Other genes we replicated have been reported to play a role in breast cancer progression such as *HOXD9* [28, 29], *TDRD10* [9, 30], *SH3PXD2A* [31] and *TSPAN15* [32], although these studies did not involve DNA methylation data as a prognostic marker. Other genes were reported in the context of other cancer types, e.g. *AHCYL2* as having a potential role in melanoma [33], colorectal [34], ovarian [35], and lung cancer [36], and *KCNS3* as potentially relevant in colon and lung cancers [37]. We also found that a CpG in *MZF1* had different survival associations in luminal A and luminal B subtypes, suggesting heterogeneity across subtypes. This CpG was independently associated with survival in luminal B tumours. *MZF1* was implicated in the progression of several other cancers, including colorectal, cervical, liver, lung, and prostate cancer (25). Future studies investigating the functional role of DNA methylation of these genes in breast tumours would contribute to elucidate their involvement in cancer progression and establish them as promising candidates for survival stratification.

## Conclusion

Our findings provide some evidence supporting the potential of DNA methylation markers to predict breast cancer outcomes. Additional larger studies are required to confirm and extend these results.

## Supplementary Information

Additional file1.

## Data Availability

The data that support the findings of this study can be made available upon reasonable request to the corresponding author.
