# Supplementary material for: Tumour DNA methylation markers associated with breast cancer survival: a replication study
Source: Breast Cancer Res. 2025 Jan 17;27:9. doi: 10.1186/s13058-024-01955-x (PMC11740461; doi:10.1186/s13058-024-01955-x)
Supplement: Supplementary file 1 — Additional file1. [file 13058_2024_1955_MOESM1_ESM.docx]

**Supplementary material**

**Table S1**. Associations of tumour DNA methylation with overall survival for 22 individual CpG sites using MCCS data (N cases= 425, N breast cancer deaths= 168)

|  | | | | Model 0 ^a^ | | Model 1 ^a^ | | Model 2 ^a^ | | Model 3 ^a^ | |  |
| --- | --- | --- | --- | --- | --- | --- | --- | --- | --- | --- | --- | --- |
| Study | CpG | Gene | Direction | HR, 95%CI | P | HR, 95%CI | P | HR, 95%CI | P | HR, 95%CI | P | Evidence of replication |
| De Almeida et al. [9] | cg01268824 | *ZNF154* | + | 1.18 (1.01,1.38) | 0.03 | 1.15 (0.98,1.34) | 0.08 | 1.07 (0.90,1.26) | 0.45 | 1.10 (0.92,1.33) | 0.30 | Yes |
|  | cg22674699 | *HOXD9* | + | 1.21 (1.03,1.41) | 0.02 | 1.17 (1.00,1.36) | 0.05 | 1.10 (0.93,1.29) | 0.27 | 1.12 (0.94,1.34) | 0.19 | Yes |
|  | cg12374721 | *C17orf93* | + | 1.18 (1.01,1.38) | 0.04 | 1.18 (1.00,1.38) | 0.05 | 1.19 (1.00,1.41) | 0.04 | 1.22 (1.02,1.46) | 0.03 | Yes |
|  | cg18081940 | *TDRD10* | + | 1.22 (1.05,1.43) | 0.01 | 1.15 (0.98,1.36) | 0.08 | 1.09 (0.92,1.29) | 0.30 | 1.13 (0.95,1.34) | 0.20 | Yes |
|  | cg04475027 | *TMEM132C* | + | 1.05 (0.90,1.22) | 0.55 | 1.00 (0.86,1.17) | 1.00 | 0.92 (0.79,1.08) | 0.29 | 0.92 (0.78,1.09) | 0.33 | No |
| Kim et al. [10] | cg03985718 | *TGFBRAP1* | - | 1.16 (0.99,1.37) | 0.07 | 1.14 (0.97,1.35) | 0.11 | 1.13 (0.95,1.34) | 0.17 | 1.13 (0.95,1.34) | 0.16 | No |
|  | cg04921068 | *PPM1L* | - | 0.92 (0.78,1.09) | 0.34 | 0.98 (0.84,1.15) | 0.84 | 0.94 (0.80,1.11) | 0.47 | 0.93 (0.78,1.10) | 0.39 | No |
|  | cg15462203 | *DVL1* | - | 1.00 (0.86,1.17) | 0.96 | 1.03 (0.87,1.20) | 0.75 | 1.05 (0.89,1.23) | 0.58 | 1.04 (0.89,1.23) | 0.60 | No |
|  | cg17827670 | *AHCYL2* | - | 0.82 (0.68,0.98) | 0.03 | 0.91 (0.76,1.07) | 0.26 | 0.87 (0.73,1.04) | 0.13 | 0.86 (0.71,1.03) | 0.11 | No |
|  | cg09926728 | *SH3PXD2A* | - | 0.88 (0.77,0.99) | 0.04 | 0.93 (0.81,1.06) | 0.29 | 0.88 (0.77,1.00) | 0.05 | 0.87 (0.76,1.00) | 0.04 | Yes |
|  | cg18703983 | *KCNS3* | - | 0.82 (0.72,0.95) | 0.01 | 0.87 (0.76,1.00) | 0.05 | 0.90 (0.77,1.05) | 0.17 | 0.90 (0.77,1.05) | 0.17 | No |
|  | cg16976520 | *ESYT2* | - | 0.89 (0.77,1.02) | 0.09 | 0.91 (0.79,1.05) | 0.20 | 0.90 (0.77,1.04) | 0.16 | 0.89 (0.77,1.04) | 0.14 | No |
|  | cg17735983 | *MZF1* | + | 1.26 (1.12,1.41) | 2×10^-4^ | 1.22 (1.08,1.37) | 0.001 | 1.17 (1.03,1.33) | 0.02 | 1.18 (1.04,1.34) | 0.01 | Yes |
|  | cg10678486 | *ELAC1* | + | 1.13 (1.00,1.28) | 0.05 | 1.10 (0.98,1.25) | 0.11 | 1.13 (1.00,1.28) | 0.06 | 1.13 (1.00,1.28) | 0.05 | No |
|  | cg13447284 |  | - | 1.11 (0.94,1.30) | 0.21 | 1.11 (0.94,0.32) | 0.22 | 1.13 (0.95,1.35) | 0.16 | 1.13 (0.95.1.34) | 0.17 | No |
|  | cg24328142 | *TSPAN15* | - | 0.91 (0.79,1.06) | 0.23 | 0.90 (0.77,1.05) | 0.17 | 0.90 (0.77,1.04) | 0.16 | 0.90 (0.77,1.05) | 0.17 | No |
|  | cg03216043 | *DNM2* | - | 1.00 (0.86,1.16) | 0.96 | 0.93 (0.79,1.08) | 0.32 | 0.90 (0.77,1.05) | 0.19 | 0.90 (0.76,1.06) | 0.22 | No |
|  | cg22776912 | *TMC3* | - | 1.08 (0.92,1.26) | 0.35 | 1.01 (0.86,1.18) | 0.93 | 1.00 (0.83,1.21) | 0.98 | 1.01 (0.83,1.22) | 0.93 | No |
|  | cg06956006 | *ACLY* | - | 1.02 (.88,1.18) | 0.80 | 0.96 (0.82,1.13) | 0.64 | 0.93 (0.79,1.10) | 0.42 | 0.93 (0.79,1.10) | 0.42 | No |
|  | cg00175150 | *ECM1* | - | 0.97 (0.84,1.12) | 0.66 | 0.96 (0.84,1.11) | 0.60 | 1.02 (0.87,1.18) | 0.84 | 1.02 (0.88,1.19) | 0.79 | No |
|  | cg15348839 |  | - | 1.05 (0.90,1.22) | 0.53 | 1.08 (0.92,1.27) | 0.36 | 1.06 (0.90,1.25) | 0.46 | 1.06 (0.90,1.25) | 0.48 | No |
|  | cg12511487 |  | - | 0.90 (0.78,1.02) | 0.17 | 0.91 (0.80,1.05) | 0.20 | 0.91 (0.78,1.05) | 0.19 | 0.91 (0.78,1.06) | 0.21 | No |

^a^ Model 0: unadjusted; Model 1: adjusted for age and country of birth; Model 2: stratified for stage (I; II; III/IV) and IHC-based subtype (luminal A, luminal B, HER2-positive, triple-negative) + adjusted for age and country of birth; Model 3: Model 2 + additional adjustment for tumour purity

**Table S2**. Associations of three tumour DNA methylation-based signatures with overall survival using MCCS data (N cases= 425, N breast cancer deaths= 168)

|  | **Overall survival; N=425; N deaths=168** | | | | | | | | |
| --- | --- | --- | --- | --- | --- | --- | --- | --- | --- |
| **Methylation signatures of survival** | **Model 0** ^a^ | | **Model 1** ^a^ | | | **Model 2** ^a^ | | **Model 3** ^a^ | |
|  | HR, 95%CI | P | HR, 95%CI | P | | HR, 95%CI | P | HR, 95%CI | P |
| Du et al. [11]; 7 CpGs | 1.05 (0.91,1.23) | 0.50 | 1.10 (0.95,1.28) | 0.20 | | 1.08 (0.92,1.27) | 0.34 | 1.07 (0.91,1.28) | 0.38 |
| Tao et al.[12]; 16 CpGs | 1.26 (1.09,1.45) | 0.002 | 1.17 (1.01,1.35) | 0.03 | | 1.12 (0.96,1.30) | 0.14 | 1.14 (0.97,1.33) | 0.11 |
| Liu et al.[15]; 28 CpGs | 1.34 (1.15,1.55) | 1.2 × 10^-4^ | 1.23 (1.05,1.43) | 0.007 | 1.14 (0.96,1.36) | | 0.12 | 1.20 (0.99,1.45) | 0.06 |

^a^ Model 0: unadjusted; Model 1: adjusted for age and country of birth; Model 2: stratified for stage (I; II; III/IV) and IHC-based subtype (luminal A, luminal B, HER2-positive, triple-negative) + adjusted for age and country of birth; Model 3: Model 2 + additional adjustment for tumour purity

**Table S3.** Heterogeneity by subtype in the association of tumour DNA methylation with overall survival, for 17 CpGs reported by Kim et al. [10] using MCCS data (N cases= 425, N breast cancer deaths= 168)

| CpG | Gene | Kim et al.  (N cases = 692, N disease-specific deaths = 87, N progress to a new tumour event = 94) | | | MCCS (N cases = 425, N all cause deaths = 168) | | | | | | |
| --- | --- | --- | --- | --- | --- | --- | --- | --- | --- | --- | --- |
|  |  |  |  |  | OS^4^ | | Heterogeneity by subtype ^5^ | | | | |
|  |  |  |  |  |  |  |  | Luminal A^6^ | | Luminal B^7^ | |
|  |  | HR, 95%CI | P | Outcome | HR, 95%CI | P | P-interaction | HR, 95%CI | P | HR, 95%CI | P |
| cg03985718 | *TGFBRAP1* | 0.48 (0.37,0.63) | 6.91×10^-8^ | OS; Any subtype^1^ | 1.13 (0.95,1.33) | 0.16 | 0.09 | 1.33 (1.05,1.68) | 0.02 | 0.86 (0.60,1.22) | 0.39 |
| cg04921068 | *PPM1L* | 0.12 (0.06-0.25) | 3.84×10^-8^ | OS; Luminal A^2^ | 0.89 (0.75,1.06) | 0.20 | 0.23 | 0.95 (0.69,1.32) | 0.78 | 0.58 (0.31,1.06) | 0.08 |
| cg15462203 | *DVL1* | 0.52 (0.41-0.66) | 4.75×10^-8^ | OS; Luminal A | 1.04 (0.89,1.22) | 0.59 | 0.69 | 1.13 (0.91,1.41) | 0.28 | 0.95 (0.68,1.33) | 0.77 |
| cg17827670 | *AHCYL2* | 0.10 (0.04-0.23) | 7.65×10^-8^ | OS; Luminal A | 0.85 (0.71,1.03) | 0.09 | 0.39 | 0.75 (0.53,1.05) | 0.10 | 0.69 (0.38,1.24) | 0.22 |
| cg09926728 | *SH3PXD2A* | 0.48 (0.37,0.63) | 6.91×10^-8^ | PFS; Any subtype^1^ | 0.87 (0.76,1.02) | 0.04 | 0.78 | 0.89 (0.75,1.04) | 0.15 | 0.85 (0.65,1.12) | 0.25 |
| cg18703983 | *KCNS3* | 0.53 (0.42-0.67) | 8.97×10^-8^ | PFS; Any subtype | 0.88 (0.76,1.02) | 0.10 | 0.33 | 1.02 (0.82,1.26) | 0.88 | 0.76 (0.60,0.95) | 0.02 |
| cg16976520 | *ESYT2* | 0.52 (0.41-0.66) | 9.62×10^-8^ | PFS; Any subtype | 0.90 (0.78,1.04) | 0.14 | 0.07 | 1.06 (0.87,1.31) | 0.55 | 0.74 (0.58,0.96) | 0.02 |
| cg17735983 | *MZF1* | 2.44 (1.78-3.35) | 3.01×10^-8^ | PFS; Luminal A^2^ | 1.18 (1.04,1.33) | 0.01 | 0.04 | 1.06 (0.89,1.25) | 0.53 | 1.33 (1.06,1.67) | 0.01 |
| cg10678486 | *ELAC1* | 1.84 (1.48-2.30) | 5.47×10^-8^ | PFS; Luminal A | 1.11 (0.98,1.26) | 0.11 | 0.16 | 1.15(0.98,1.34) | 0.09 | 1.12 (0.93,1.35) | 0.23 |
| cg13447284 |  | 0.48 (0.37-0.63) | 8.11×10^-8^ | PFS; Luminal A | 1.13 (0.96,1.34) | 0.15 | 0.58 | 1.26 (1.00,1.59) | 0.05 | 0.96 (0.69,1.34) | 0.82 |
| cg24328142 | *TSPAN15* | 0.33 (0.22-0.49) | 3.04×10^-8^ | PFS; Luminal B^2^ | 0.88 (0.76,1.03) | 0.11 | 0.02 | 0.96 (0.79,1.18) | 0.73 | 0.63 (0.47,0.68) | 0.003 |
| cg03216043 | *DNM2* | 0.15 (0.08-0.29) | 3.87×10^-8^ | PFS; Luminal B | 0.88 (0.74,1.04) | 0.12 | 0.57 | 0.95 (0.76,1.18) | 0.65 | 0.83 (0.59,1.15) | 0.26 |
| cg22776912 | *TMC3* | 0.17 (0.09-0.32) | 5.85×10^-8^ | PFS; Luminal B | 1.03 (0.86,1.24) | 0.72 | 0.30 | 1.12 (0.85,1.48) | 0.41 | 0.68 (0.43,1.07) | 0.09 |
| cg06956006 | *ACLY* | 0.14 (0.07-0.29) | 9.47×10^-8^ | PFS; Luminal B | 0.92 (0.78,1.09) | 0.33 | 0.55 | 1.03 (0.82,1.29) | 0.83 | 0.82 (0.58,1.16) | 0.25 |
| cg00175150 | *ECM1* | 0.49 (0.39-0.63) | 1.32×10^-8^ | PFS; Post-menopause^3^ | 1.01 (0.87,1.18) | 0.85 | 0.08 | 1.13 (0.93,1.38) | 0.22 | 0.69 (0.49,0.99) | 0.04 |
| cg15348839 |  | 0.53 (0.43-0.67) | 4.19×10^-8^ | PFS; Post-menopause | 1.07 (0.91,1.25) | 0.43 | 0.53 | 1.13 (0.92,1.39) | 0.23 | 0.86 (0.62,1.19) | 0.35 |
| cg12511487 |  | 0.39 (0.28-0.55) | 8.23×10^-8^ | PFS; Post-menopause | 0.91 (0.79,1.06) | 0.23 | 0.04 | 0.95 (0.78,1.6) | 0.61 | 0.69 (0.52,0.93) | 0.01 |

OS, overall survival; PFS, progression-free survival

1. Adjusted for age + race + stage + menopause status + tumour purity + cell type proportion
2. Adjusted for age + race + stage + menopause status + tumour purity + cell type proportion + gene expression-based subtype × CpG site
3. Adjusted for age + race + stage + menopause status + tumour purity + cell type proportion + menopausal status × CpG site
4. Adjusted for age + country of birth + tumour stage groups + tumour purity + stratified by IHC-based subtype
5. Adjusted for age + country of birth + tumour stage groups + tumour purity + IHC-based subtype × CpG site
6. Luminal A: N cases = 242, N deaths = 101
7. Luminal B: N cases = 87, N deaths = 32
